# External validation of the P-Score in the prospective, pre-biopsy DEPROMP cohort: integrated mpMRI and PSMA PET/CT risk stratification for prostate cancer detection

**DOI:** 10.1007/s00259-026-08073-0

**Published:** 2026-07-16

**Authors:** Clarissa Julia Schmidt, Florian C Gaertner, Barbara Kreppel, Markus Essler, Julian A Luetkens, Ulrike Attenberger, Michael Anspach, Carsten-Henning Ohlmann, Marit Bernhardt, Glen Kristiansen, Stefan Hauser, Jörg Ellinger, Manuel Ritter, Philipp Krausewitz

**Affiliations:** 1 University Hospital Bonn, Department of Urology and Paediatric Urology, Bonn, North Rhine-Westphalia Germany; 2 University Hospital Bonn, Department of Nuclear Medicine, Bonn, North Rhine- Westphalia Germany; 3 University Hospital Bonn, Department of Diagnostic and Interventional Radiology, Bonn, North Rhine-Westphalia Germany; 4 Department of Radiology and Nuclear Medicine, Medical University of Vienna, Vienna, Austria; 5 Johanniter Hospital Bonn, Department of Urology, Bonn, North Rhine-Westphalia Germany; 6 University Hospital Bonn, Institute of Pathology, Bonn, North Rhine-Westphalia Germany; 7 Department of Urology, GFO Hospital Troisdorf, Troisdorf, North Rhine-Westphalia Germany

**Keywords:** Prostate cancer, P-Score, Multiparametric MRI, PSMA-PET/CT, Diagnostic accuracy, Risk stratification

## Abstract

**Purpose:**

This study aimed to externally validate the P-Score, a composite scoring system combining PI-RADS and PRIMARY score, for its diagnostic accuracy in detecting clinically significant (ISUP ≥ 2) and higher-grade (ISUP ≥ 3) prostate cancer (PCa).

**Methods:**

230 biopsy-naïve men with suspected PCa were prospectively enrolled in the DEPROMP trial and underwent multiparametric MRI and [^68^Ga]Ga-DKFZ-PSMA-11 PET/CT. Lesions were assessed using PI-RADS v2.1, the PRIMARY score, and the derived P-Score. Targeted biopsies were guided by imaging findings, and histopathology served as the reference standard. Diagnostic performance was evaluated using receiver operating characteristic curve analyses and DeLong’s test to compare the area under the curve (AUC) between scoring systems.

**Results:**

For clinically significant PCa (ISUP ≥ 2), no significant difference in diagnostic performance was observed between the P-Score (AUC 0.796) and PI-RADS (AUC 0.804; *p* = 0.72), whereas the P-Score significantly outperformed the PRIMARY score (AUC 0.730; *p* < 0.001). Similarly, for detection of ISUP ≥ 3 disease, no significant difference in diagnostic performance was observed between the P-Score (AUC 0.840) and PI-RADS (AUC 0.830; *p* = 0.66). In the PI-RADS 3 subgroup, diagnostic performance remained limited, with AUCs of 0.573 (95% CI: 0.407–0.739) for the P-Score and 0.556 (95% CI: 0.389–0.722) for the PRIMARY score. Overall, the P-Score demonstrated robust diagnostic performance across disease categories and showed comparable accuracy to PI-RADS.

**Conclusion:**

The P-Score demonstrated diagnostic performance comparable to PI-RADS in this external validation cohort. While providing a standardized framework integrating anatomical and molecular imaging findings, further studies are warranted to determine whether it has a role in selected clinical scenarios and prospective research settings.

**Supplementary Information:**

The online version contains supplementary material available at https://doi.org/10.1007/s00259-026-08073-0.

## Introduction

Prostate cancer (PCa) remains one of the most common malignancies in men worldwide, with accurate detection and risk stratification being central to effective clinical management [1]. Multiparametric Magnetic Resonance Imaging (mpMRI) has become a cornerstone in PCa diagnostics, standardized through the the Prostate Imaging Reporting and Data System (PI-RADS). Despite its widespread use, mpMRI alone shows limitations in reliably identifying clinically significant PCa (csPCa) (ISUP ≥ 2), particularly in equivocal cases, leading to both under- and overdiagnosis by unnecessary biopsies and missed diagnoses [2, 3]. In the last decade, Prostate-specific membrane antigen positron emission tomography/computed tomography (PSMA-PET/CT) has emerged as a complementary imaging modality. The PRIMARY score and the Prostate Cancer Molecular Imaging Standardized Evaluation (PROMISE) miTNM (molecular imaging TNM system) framework have introduced structured approaches to interpreting local findings of PSMA-PET/CT imaging, enabling improved reproducibility and standardization [4–6]. Importantly, PROMISE has demonstrated not only diagnostic utility but also prognostic value by predicting biochemical recurrence following radical prostatectomy, and recent multicenter evidence shows that the updated PROMISE–based nomograms derived from PSMA-PET/CT provide independent and accurate stratification of overall survival at initial staging [7, 8]. Studies further highlight that the combination of mpMRI and PSMA-PET/CT significantly improves intraprostatic tumor localisation and csPCa detection compared to either modality alone [9–11]. As the clinical use of dual imaging expands, the need for an integrated classification system becomes evident. In response, Emmett et al. proposed the P-Score, combining PI-RADS and the PRIMARY score into a unified structured diagnostic tool [6, 12–14]. To our knowledge, this study represents the first external validation of the P-Score in an independent prospective phase II cohort. We therefore aimed to assess its diagnostic accuracy and clinical utility and to evaluate its potential role in multimodal prostate cancer imaging.

## Materials and methods

### Study population

This validation cohort comprises 230 patients who underwent both [^68^Ga]Ga-DKFZ-PSMA-11 PET/CT and mpMRI as part of the DEtection rate of clinically significant PROstate cancer by mpMRI and PSMA-PET/CT fusion biopsy (DEPROMP) trial. The DEPROMP Trial is a prospective, open-label, phase II interventional study. Patients were prospectively screened for eligibility at the participating institutions based on clinical suspicion of localized prostate cancer. Inclusion criteria comprised: (1) suspected localized prostate cancer based on repeated PSA values ≥ 4 ng/ml, suspicious digital rectal examination, and/or tumor-suspicious findings on transrectal ultrasound; (2) age ≥ 45 and < 76 years; (3) no previous prostate biopsy; and (4) ability to provide written informed consent and comply with study procedures. Exclusion criteria were allergy to the radiopharmaceutical ^68^Ga-PSMA or related compounds, abuse of medications/drugs/alcohol, PSA levels > 100 ng/ml with suspected advanced metastatic prostate cancer, and severe chronic renal impairment defined as estimated creatinine clearance < 30 ml/min, according to the published DEPROMP study protocol [15]. Between March 2021 and January 2023, 246 patients were recruited at two participating institutions, of whom 16 withdrew before biopsy, resulting in a final cohort of 230 patients included in the present analysis [16]. A study flowchart illustrats patient screening, eligibility assessment, exclusions, withdrawals, and final cohort composition as supplementray Fig. S1. mpMRI scans were independently interpreted by experienced uro-radiologists blinded to the corresponding PSMA PET/CT findings, while PSMA PET/CT scans were independently interpreted by experienced nuclear medicine physicians blinded to the corresponding mpMRI results in accordance with the DEPROMP study protocol.Comprehensive protocol details were previously published [15]. An analysis of the sensitivity, specificity, positive predictive value, negative predictive value, and diagnostic accuracy for PCa and csPCa of mpMRI, PSMA-PET/CT, prostate biopsy modalities, and the respective imaging classifications (PI-RADS v2.1 classification, PROMISE miTNM classification v1.0, molecular imaging prostate-specific membrane antigen (miPSMA) score) was part of the initial study protocol [15, 16]. For the current study, the PRIMARY score and the consecutive P-Score were assessed post hoc. The German National Federal Office for Radiation Protection granted ethics committee approval (ZD 3-22464/2020-107-G).

### mpMRI: protocol and assessment

mpMRI was performed as part of routine care using PI-RADS v2.1, including axial, coronal, and sagittal T2-weighted, DWI, and dynamic contrast-enhanced T1-weighted sequences. Two experienced uro-radiologists independently reviewed the scans, blinded to PSMA-PET/CT findings. Lesions with PI-RADS ≥ 3 were analyzed for location, size, enhancement, and ADC values, in accordance with the DEPROMP study protocol [15].

### PSMA-PET/CT: protocol and assessment

All imaging was conducted at the Department of Nuclear Medicine, University Hospital Bonn. [^68^Ga]Ga-DKFZ-PSMA-11 (HBED-CC) was synthesized on-site under good laboratory practice and administered at 2 MBq per kilogram body weight. After an uptake period of 50 to 100 min, patients underwent PSMA-PET imaging with a low-dose, non-contrast-enhanced CT scan. Two experienced nuclear medicine physicians independently evaluated the scans according to the PROMISE miTNM version 1.0 framework [5], blinded to mpMRI results. PROMISE miTNM version 1.0 was used in accordance with the original DEPROMP study protocol, as it represented the current standard at the time of study conception in 2019 [15], and to maintain consistency with the PRIMARY and P-Score frameworks proposed by Emmett et al. [12]. Intraprostatic PSMA uptake was retrospectively assessed using the PRIMARY score, which incorporates uptake intensity and zonal anatomy, recorded using a study-specific prostate diagram adapted from the PI-RADS version 2.1 sector map [6]. For each suspicious lesion, the maximum standardized uptake value (SUVmax) was also documented, in accordance with the DEPROMP study protocol [15].

### P-score

The P-Score was calculated according to the framework proposed by Emmett et al. by integrating PI-RADS v2.1 and PRIMARY scores into a composite 5-point risk stratification system [12]. The allocation of P-Score categories based on PI-RADS and PRIMARY score combinations is summarized in Table 1.

**Table 1:** Derivation of the P-Score based on combinations of PI-RADS and PRIMARY scores according to the framework proposed by Emmett et al. [12]

PRIMARY score	PI-RADS 1–2	PI-RADS 3	PI-RADS 4	PI-RADS 5
1	P-Score 1	P-Score 2	P-Score 3	P-Score 3
2	P-Score 2	P-Score 2	P-Score 3	P-Score 3
3	P-Score 3	P-Score 4	P-Score 4	P-Score 4
4	P-Score 3	P-Score 4	P-Score 4	P-Score 4
5	P-Score 5	P-Score 5	P-Score 5	P-Score 5

### Prostate biopsy

Standardized transrectal biopsy procedures followed the DEPROMP trial protocol using a pre-configured template and a software-assisted fusion system (KOELIS Trinity^®^), as detailed in the study protocol [15]. Targeted biopsies were performed in regions suspicious on mpMRI (PI-RADS ≥ 3) and/or PSMA-PET/CT (PSMA expression score ≥ 1), selecting up to three lesions per patient. All specimens were assessed histologically in accordance with established guidelines at the Institute of Pathology of the University of Bonn, a national tertiary reference center for genitourinary pathology [17, 18]. Biopsy specimens were labeled based on their anatomical origin based on the PI-RADS v2.1 sector map [19] and the highest Gleason score was recorded [15]. Imaging-pathology correlation was performed on a lesion-based level using the PI-RADS v2.1 sector map as a common anatomical reference framework for mpMRI, PSMA PET/CT, and targeted biopsy localization. Suspicious lesions identified on mpMRI and/or PSMA PET/CT were graphically annotated on a PI-RADS v2.1 derived sector map and targeted using software-assisted fusion biopsy guidance (KOELIS Trinity^®^). In addition, all patients underwent an institutional standardized systematic biopsy of the peripheral zone [15]. Histopathological specimens were documented according to anatomical sector localization and biopsy modality (systematic, MRI-targeted, or PSMA-targeted biopsy). Correlation between imaging findings and histopathology was therefore based on sector localization and targeted biopsy documentation [15]. Nevertheless, exact spatial correlation between imaging findings and biopsy samples remains inherently limited due to differences in imaging resolution and biopsy sampling precision.

### Statistical analysis

To assess the diagnostic performance of the P-Score, PRIMARY score, and PI-RADS in detecting csPCa, receiver operating characteristic (ROC) curve analyses were performed and the corresponding area under the curve (AUC) values were calculated. In line with the classification applied by Emmett et al., csPCa was defined as International Society of Urological Pathology (ISUP) grade group ≥ 2, while higher-grade disease was further specified as ISUP grade group ≥ 3 [13]. This stratification reflects the increasing clinical importance of more aggressive PCa subtypes and enables a refined evaluation of diagnostic accuracy across risk categories. Comparative analyses of AUCs between scoring systems were conducted using DeLong’s test for correlated ROC curves. ROC analyses were performed using the full ordinal score ranges of PI-RADS, PRIMARY score, and P-Score as ordinal predictors without predefined dichotomization thresholds. For clinical interpretation, scores ≥ 3 in the respective scoring systems were considered suspicious for malignancy, whereas scores 1–2 were regarded as low-risk/negative findings. All statistical analyses and data visualizations were performed using RStudio (version 2024.09.1 + 394 for Windows), with a significance level of p < 0.05 considered statistically significant.

## Results

### Study Population

The characteristics of the participants (*n* = 230) participating in the DEPROMP trial are summarized in Table 2, as previously published [16].

**Table 2:** Baseline clinical, imaging, and histopathological characteristics of the study population (*n* = 230) as previously published by Krausewitz et al. Data are presented as median (interquartile range) or number (percentage), as appropriate. PI-RADS and miPSMA scores were assigned based on MRI and PSMA-PET/CT findings, respectively. ISUP grade group and D’Amico risk classification were derived from histopathological analysis of targeted biopsy specimens. *Patients with missing data: SUVmax (*n* = 8). In these 8 patients, no intraprostatic lesion was detectable (miT0); consequently, no SUVmax was measured due to the absence of a target lesion [16]

Variable	Median (Interquartile range) or *n* (%)
**Age at biopsy (yr.)**	63.0 (59–68)
**PSA (ng/ml)**	6.9 (5.6–9.8)
**PI-RADS Score**	
1/2	91 (39.6)
3	43 (18.7)
4	47 (20.4)
5	49 (21.3)
**PRIMARY score**	
1	6 (2.6)
2	73 (31.7)
3	17 (7.4)
4	100 (43.5)
5	34 (14.8)
SUVmax*	6.0 (5.0-8.5)
**miPSMA Score**	
0	8 (3.5)
1	75 (32.6)
2	126 (54.8)
3	21 (9.1)
**ISUP Grade Group**	
Benign findings	93 (40.4)
1	25 (10.9)
2	54 (23.5)
3	33 (14.3)
4	12 (5.2)
5	13 (5.7)
**D’Amico risk group**	
low	21 (9.1)
intermediate	81 (35.2)
high	35 (15.2)

(Figure 1) illustrates the percentage distribution of ISUP grade groups across the five P-Score categories within the study population. A clear trend is observed, with higher P-Scores being associated with an increased proportion of patients diagnosed with csPCa (ISUP ≥ 2). In contrast, lower P-Scores (particularly 1 and 2) are predominantly associated with benign findings or low-risk disease (ISUP 1).

**Fig. 1:**
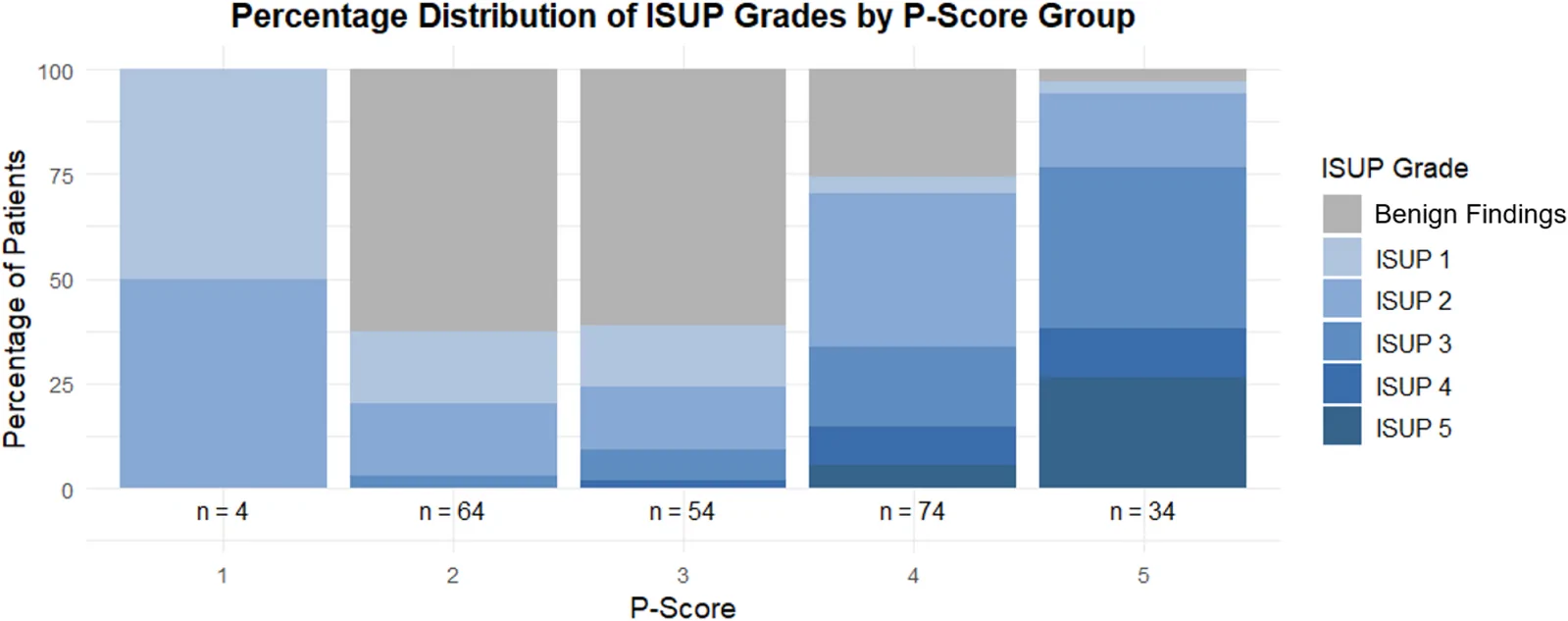
Illustrating the percentage distribution of ISUP grade groups across the five P-Score categories. The total number of patients in each P-Score group is indicated below the respective bars

### Study findings

**Fig. 2:**
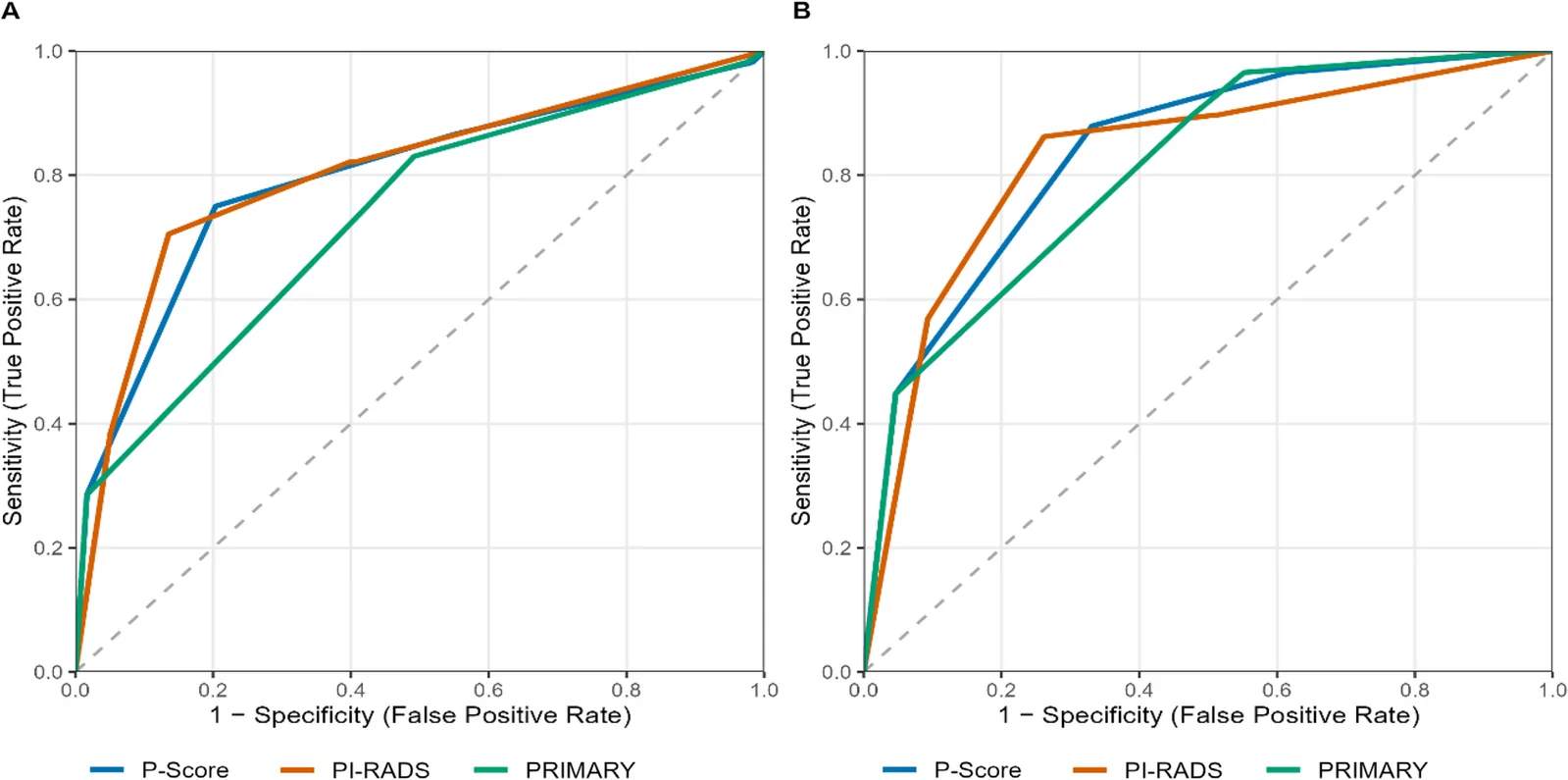
ROC for the detection of csPCa (ISUP ≥ 2) using P-Score, PRIMARY score, and PI-RADS

For the detection of csPCa (ISUP ≥ 2), the P-Score demonstrated an AUC of 0.796 (95% CI: 0.739–0.853). The PI-RADS score showed a slightly higher AUC of 0.804 (95% CI: 0.747–0.860), while the PRIMARY score achieved a lower AUC of 0.730 (95% CI: 0.670–0.790), Table 3; (Fig. 2A). DeLong’s test revealed a statistically significant difference between the P-Score and PRIMARY score (AUC difference = − 0.066, 95% CI: − 0.099 to − 0.034, *p* < 0.001), indicating superior performance of the P-Score. In contrast, no significant difference was observed between P-Score and PI-RADS (AUC difference = + 0.008, 95% CI: − 0.035 to + 0.050, *p* = 0.72). For detection of higher-grade disease (ISUP ≥ 3), the P-Score showed the highest AUC at 0.840 (95% CI: 0.786–0.894), followed by PI-RADS at 0.830 (95% CI: 0.768–0.893) and the PRIMARY score at 0.811 (95% CI: 0.755–0.866) (Table 3; Fig. 2B). Compared with the P-Score, no statistically significant difference was observed for the PRIMARY score (ΔAUC = − 0.029, 95% CI: − 0.060 to + 0.001, *p* = 0.061). No significant difference was found between P-Score and PI-RADS (ΔAUC = − 0.010, 95% CI: − 0.053 to + 0.033, *p* = 0.66). Overall, the P-Score demonstrated diagnostic performance comparable to PI-RADS, while significantly outperforming the PRIMARY score for detection of ISUP grade group ≥ 2 disease.

Figure 2B: ROC for the detection of ISUP grade group ≥ 3 PCa using P-Score, PRIMARY score, and PI-RADS.

Subgroup analysis of patients with PI-RADS 3 lesions showed AUCs of 0.573 (95% CI: 0.407–0.739) for the P-Score and 0.556 (95% CI: 0.389–0.722) for the PRIMARY score, without a statistically significant difference between both scoring systems(Table 3). The difference between the two scores was not statistically significant (ΔAUC = − 0.017, 95% CI: − 0.079 to + 0.045, *p* = 0.582), indicating comparable diagnostic value within the PI-RADS 3 cohort. Within the PI-RADS 3 subgroup (*n* = 44), 23 patients (52.3%) were up-staged by the P-Score, including 8/23 patients (34.8%) with csPCa, whereas 18 patients (40.9%) were down-staged, with absence of csPCa in 14/18 patients (77.8%). Overall, correct down-staging occurred in 14/44 patients (31.8%), while correct up-staging was observed in 8/44 patients (18.2%) (Supplementary Table S4).

Threshold-based analyses for PI-RADS, PRIMARY score, and P-Score using cutoffs of ≥ 3 and ≥ 4 are provided in Supplementary Table S1 and Supplementary Fig. S2. Using a threshold of ≥ 3, the P-Score demonstrated the highest csPCa detection rate (86.6%) with the lowest missed csPCa rate (13.4%), compared with PI-RADS (82.1% detection; 17.9% missed csPCa) and the PRIMARY score (83.0% detection; 17.0% missed csPCa). At a threshold of ≥ 4, csPCa detection rates decreased for all scoring systems, with detection rates of 70.5% for PI-RADS, 75.0% for the PRIMARY score, and 75.0% for the P-Score, corresponding to missed csPCa rates of 29.5%, 25.0%, and 25.0%, respectively. Sensitivity, specificity, positive predictive value, and negative predictive value for csPCa detection using thresholds of ≥ 3 and ≥ 4 for PI-RADS, PRIMARY score, and P-Score are summarized in Supplementary Table S2. The distribution of histopathological ISUP grade groups across PI-RADS and PRIMARY score categories is illustrated in Supplementary Figs. S3 and S4. Supplementary analyses of individual P-Score strata demonstrated increasing prevalence of csPCa across higher P-Score categories. Within the combined low-risk P-Score 1–2 category, csPCa was present in 15/68 patients (22.1%), corresponding to a non-csPCa rate of 77.9% whereas the prevalence of csPCa reached 94.1% in patients with a P-Score of 5 (Supplementary Table S3).

**Table 3:** AUCs with 95% confidence intervals, AUC differences versus P-Score, and corresponding p-values for the detection of csPCa (ISUP ≥ 2); ISUP ≥ 3 and subgroup PI-RADS 3 using P-Score, PRIMARY, and PI-RADS

Metric	*P*-Score	PRIMARY	PI-RADS
AUC for ISUP ≥ 2 [95% CI]	0.796 [0.739–0.853]	0.730 [0.670–0.790]	0.804 [0.747–0.860]
Difference to P-Score (ISUP ≥ 2)	–	-0.066 [–0.099, − 0.034] *p* < 0.001	+ 0.008 [–0.035, + 0.050] *p* = 0.72
AUC for ISUP ≥ 3 [95% CI]	0.840 [0.786–0.894]	0.811 [0.755–0.866]	0.830 [0.768–0.893]
Difference to P-Score (ISUP ≥ 3)	–	-0.029 [–0.060, + 0.001] *p* = 0.061	-0.010 [–0.053, + 0.033] *p* = 0.66
AUC for PI-RADS 3 [95% CI]	0.573 [0.407–0.739]	0.556 [0.389–0.722]	–
Difference to P-Score (PI-RADS 3)	–	–0.017 [–0.079, + 0.045] *p* = 0.58	–

## Discussion

In this external validation study, the P-Score demonstrated diagnostic performance comparable to PI-RADS for the detection of clinically significant prostate cancer (ISUP ≥ 2) and higher-grade disease (ISUP ≥ 3), while significantly outperforming the PRIMARY score for the detection of clinically significant prostate cancer. Increasing P-Score categories were associated with higher histopathological tumor grade distribution (Fig. 1). However, no statistically significant differences in diagnostic performance were observed between the P-Score and PI-RADS. For the detection of csPCa defined as ISUP grade group ≥ 2, the P-Score significantly outperformed the PRIMARY score and showed comparable diagnostic accuracy to PI-RADS. While the PRIMARY score alone has limited standalone value, its integration into the P-Score substantially enhanced clinical utility, consistent with previous findings [6]. From a clinical perspective, the P-Score may improve precision in identifying patients who truly require prostate biopsy, thereby reducing overdiagnosis while maintaining reliable detection of clinically relevant disease [20]. This observation aligns with decision curve analyses demonstrating that combined imaging strategies provide higher net clinical benefit than biopsy alone [9, 11]. Our findings are consistent with reports by Emmett et al., showing that although differences between the P-Score and PI-RADS may not reach statistical significance, integrated imaging approaches offer robust diagnostic performance with straightforward interpretability [12].

For higher-grade PCa defined as ISUP grade group ≥ 3, the P-Score demonstrated the highest diagnostic accuracy among the evaluated approaches and showed a numerical advantage over the PRIMARY score [12]. This finding suggests that the P-Score is particularly effective in identifying aggressive tumors that are most likely to benefit from timely intervention and treatment. The integration of PSMA PET/CT information via the PRIMARY score with mpMRI appears to enhance detection of high-grade disease and reinforces the clinical value of the P-Score in PCa diagnostics [21]. The higher area under the curve observed for ISUP grade group ≥ 3 is consistent with results from the original P-Score development cohort and supports its potential role in reducing underdiagnosis of aggressive tumors [12].

Although the addition of PSMA PET/CT may be considered particularly relevant in equivocal cases such as PI-RADS 3 lesions, where mpMRI alone has known limitations [22], the P-Score did not demonstrate clear superiority in this subgroup within our cohort. Accordingly, despite recently demonstrated overall improvements in diagnostic value, the benefits of combined imaging in difficult-to-assess PI-RADS 3 lesions remain limited [9, 16]. We reported previously a modest additional diagnostic yield of PSMA-PET/CT in patients with mpMRI-negative or equivocal findings corresponding to PI-RADS categories 1–3, with csPCa detected in 22% of men [16]. Similarly, Shi et al. demonstrated high diagnostic accuracy of [^68^Ga]PSMA PET/MRI and reported added clinical value in PI-RADS 3 lesions through improved risk stratification and reduction of unnecessary biopsies [23]. In contrast, within our PI-RADS 3 subgroup, both the P-Score and the PRIMARY score showed limited performance for the detection of csPCa, with no significant difference between them. Although a numerically higher area under the curve for the P-Score may suggest a slight incremental value of combining mpMRI and PSMA PET/CT, overall diagnostic accuracy remained suboptimal, underscoring the need for further validation in larger and well-characterized cohorts. These findings are supported by earlier studies reporting potential benefits of PSMA PET/MRI in refining lesion characterization in intermediate-risk disease [21, 23].

### Contextualization with previous literature and study limitations

The limited performance observed in equivocal lesions is most plausibly explained by the restricted specificity of PSMA-based imaging for intraprostatic findings, which constrains its added value within a score-based framework. Differences between our results and previously reported high diagnostic accuracy may further be explained by variation in study populations. Emmett et al. reported areas under the curve approaching 0.9 in enriched cohorts with a high prevalence of clinically significant and high-grade prostate cancer and acknowledged selection bias and expert multi-reader assessment as contributing factors to increased diagnostic performance [12]. Compared with the validation cohort reported by Emmett et al., our DEPROMP cohort showed a lower prevalence of csPCa, whereas fewer patients were assigned to P-Score 1. This apparent discrepancy may be explained by differences in cohort composition, study design, and imaging score distribution. According to the P-Score framework, P-Score 1 is assigned only in cases with concordant low-risk findings, specifically PI-RADS 1–2 combined with PRIMARY score 1. In our cohort, PRIMARY score 1 was rare, occurring in only 6 of 230 patients, thereby limiting the number of possible P-Score 1 assignments. In contrast, the validation cohort reported by Emmett et al. included a higher prevalence of csPCa and was retrospectively accrued, with the authors acknowledging selection bias and overrepresentation of high-grade malignancy. Therefore, differences in referral patterns, reader assessment, and the distribution of low-risk PSMA PET findings may have contributed to the lower frequency of P-Score 1 in our cohort. Finally, the very small number of patients classified as P-Score 1 in DEPROMP limits robust interpretation of histopathological distributions within this subgroup, including the absence of benign findings. Although all imaging examinations were interpreted independently by experienced readers using standardized PI-RADS and PRIMARY criteria, inter-reader variability, particularly for PI-RADS assessment, remains a known limitation and may have influenced diagnostic performance estimates. This factor may partially contribute to differences between our findings and previously published cohorts. Shi et al. prospectively evaluated a strictly reviewed PI-RADS 3 population and reported high accuracy despite a low prevalence of clinically significant disease and limited event numbers [23]. In contrast, the lower areas under the curve observed in our cohort likely reflect a real-world spectrum dominated by diagnostically challenging equivocal lesions and a lower proportion of unequivocally high-grade tumors, resulting in reduced but more generalizable performance. Another potential limitation relates to the variability in PSMA PET acquisition timing. While the original PRIMARY study protocol by Emmett et al. recommended imaging acquisition at a minimum of 60 min after injection of [^68^Ga]Ga-PSMA-11 [13], the DEPROMP study permitted a broader acquisition window of 50–100 min post injection. Although previous dynamic imaging data suggest clinically acceptable lesion visualization across this interval, differences in uptake time may influence intraprostatic tracer uptake and lesion conspicuity, potentially affecting PRIMARY score and P-Score assessment. In addition to SUVmax, future studies may also evaluate the potential role of alternative lesion-based quantitative PET parameters, such as intraprostatic SUVmean, for refinement of structured PSMA PET-based risk stratification approaches.

While the standalone applicability of mpMRI and PSMA-PET/CT is promising, the added value of their combination remains to be fully defined. Meta-analytic evidence indicates that improvements in sensitivity and specificity over mpMRI alone are limited, with comparable diagnostic accuracy reported for both modalities, consistent with our findings [24]. In this context, it should be emphasized that the P-Score is not an independent diagnostic test but a structured framework integrating information derived from PI-RADS and the PRIMARY score. Accordingly, the present study was designed to externally validate the recently proposed P-Score framework in an independent prospective cohort. As no statistically significant improvement over PI-RADS was observed, routine implementation of P-Score assessment in all biopsy-naïve patients cannot be recommended based on the present findings. However, when both mpMRI and PSMA PET/CT have already been acquired as part of the diagnostic work-up, the P-Score may facilitate standardized multimodal interpretation in certain scenarios. Further prospective studies are needed to determine in which cases the P-Score provides additional clinical benefit. In addition to radiation exposure and examination costs, reimbursement and accessibility of PSMA PET/CT remain important barriers to clinical adoption [25]. However, the P-Score may still provide value in selected clinical scenarios, including higher pre-test probability of clinically significant disease, or within prospective clinical trial settings evaluating imaging-guided biopsy pathways and treatment stratification. Current prostate cancer diagnostic pathways are increasingly evolving toward personalised and risk-adapted approaches integrating imaging findings with additional biomarkers and clinical risk stratification tools. In this context, the P-Score may represent a structured multimodal imaging framework that could support decision-making in selected clinical scenarios, particularly heterogeneous risk profiles. Similar to recently proposed personalised diagnostic strategies combining MRI with complementary biomarkers and imaging modalities, integrated mpMRI and PSMA PET/CT assessment may contribute to improved patient selection for biopsy and reduction of unnecessary procedures [26]. However, further prospective studies are required to define the optimal role of the P-Score within future prostate cancer diagnostic workflows. These considerations are in line with the original P-Score framework proposed by Emmett et al., who also emphasized the importance of further validation and cost-effectiveness evaluation prior to broader implementation. Nevertheless, integration of the P-Score may support the establishment of a standardized and quality-assured interpretation framework analogous to PI-RADS and PRIMARY, facilitating unified assessment [27, 28]. Beyond diagnostic applications, structured PSMA-PET/CT-based assessment systems may also provide prognostic information. Recently, Karpinski et al. demonstrated in a large international multicenter PROMISE registry cohort that a PRIMARY score of 5 on initial staging PSMA-PET/CT was independently associated with shorter overall survival and provided additive prognostic value when combined with PI-RADS or clinical T stage [29]. These findings further support the broader clinical relevance of structured PSMA-PET/CT-derived assessment systems beyond primary cancer detection. Although evaluation of long-term oncologic outcomes was beyond the scope of the DEPROMP trial, future studies should investigate whether integrated multimodal scoring systems such as the P-Score may similarly provide prognostic value and contribute to outcome prediction. However, PI-RADS 3 lesions continue to represent a major diagnostic challenge due to imaging heterogeneity and low prevalence of clinically significant disease, highlighting the need for improved stratification tools integrating imaging, clinical, and genomic data.

In this external validation of the P-Score we could demonstrate a high diagnostic accuracy for clinically significant PCa (ISUP ≥ 2) by the combined imaging classification, with special emphasis on the detection of ISUP 3 PCa. In this external validation cohort, the P-Score demonstrated diagnostic performance comparable to PI-RADS while significantly outperforming the PRIMARY score for the detection of clinically significant prostate cancer. The P-Score provides a standardized framework integrating anatomical and molecular imaging findings. Further prospective studies are required to determine its clinical role and potential utility within multimodal prostate cancer diagnostic pathways. 

## Supplementary Information

Below is the link to the electronic supplementary material.

Supplementary Material 1

## Data Availability

All data generated or analyzed regarding the Concordance of Prostate Cancer Extent between MRI and PSMA-PET/CT is included in this article. Further inquiries can be directed at the corresponding author.

## Abbreviations

AUC: area under the curve
csPCa: clinically significant prostate cancer
DEPROMP Trial: DEtection rate of clinically significant PROstate cancer by mpMRI and PSMA-PET/CT fusion biopsy Trial
[68Ga]Ga-DKFZ-PSMA-11: [68Ga]Ga-DKFZ-PSMA-11 positron emission tomography/computed tomography
ISUP: International Society of Urological Pathology
miPSMA: miPSMAMolecular imaging prostate-specific membrane antigen
miTNM: molecular imaging TNM system
mpMRI: Multiparametric magnetic resonance imaging
MRI: Magnetic Resonance Imaging
PCa: Prostate cancer
PI-RADS: Prostate Imaging Reporting and Data System
PROMISE: Prostate Cancer Molecular Imaging Standardized Evaluation
PSA: Prostate-specific antigen
PSMA: Prostate-specific membrane antige
PSMA-PET/CT: Prostate-specific membrane antigen positron emission tomography/computed tomography
ROC: receiver operating characteristic
SUVmax: Maximum standardized uptake value
